# Mortality amongst Patients with Influenza-Associated Severe Acute Respiratory Illness, South Africa, 2009-2013

**DOI:** 10.1371/journal.pone.0118884

**Published:** 2015-03-18

**Authors:** Cheryl Cohen, Jocelyn Moyes, Stefano Tempia, Michelle Groome, Sibongile Walaza, Marthi Pretorius, Halima Dawood, Meera Chhagan, Summaya Haffejee, Ebrahim Variava, Kathleen Kahn, Anne von Gottberg, Nicole Wolter, Adam L. Cohen, Babatyi Malope-Kgokong, Marietjie Venter, Shabir A. Madhi

**Affiliations:** 1 Centre for Respiratory Diseases and Meningitis, National Institute for Communicable Diseases of the National Health Laboratory Service, Johannesburg, South Africa; 2 School of Public Health, Faculty of Health Sciences, University of the Witwatersrand, Johannesburg, South Africa; 3 Influenza Division, Centers for Disease Control and Prevention, Atlanta, Georgia, United States of America; 4 Influenza Programme, Centers for Disease Control and Prevention—South Africa, Pretoria, South Africa; 5 Medical Research Council, Respiratory and Meningeal Pathogens Research Unit, Faculty of Health Sciences, University of the Witwatersrand, Johannesburg, South Africa; 6 Department of Science and Technology/National Research Foundation: Vaccine Preventable Diseases; University of the Witwatersrand; Johannesburg; South Africa; 7 School of Pathology, Faculty of Health Sciences, University of the Witwatersrand, Johannesburg, South Africa; 8 Zoonosis Research Unit, Department of Medical Virology, University of Pretoria; 9 Department of Medicine, Klerksdorp Tshepong Hospital; 10 Department of Medicine, Faculty of Health Sciences, University of the Witwatersrand, Johannesburg, South Africa; 11 MRC/Wits Rural Public Health and Health Transitions Research Unit (Agincourt), School of Public Health, Faculty of Health Sciences, University of the Witwatersrand, Johannesburg, South Africa; 12 Centre for Global Health Research, Umeå University, Umeå, Sweden; 13 INDEPTH Network, Accra, Ghana; 14 Department of Paediatrics, University of KwaZulu Natal, Durban, South Africa; 15 Department of Medicine, Pietermaritzburg Metropolitan Hospital, Pietermaritzburg, South Africa; 16 Department of Medicine, University of KwaZulu Natal, Durban, South Africa; 17 School of Pathology, University of KwaZulu Natal, Durban, South Africa; 18 Global Disease Detection, United States Centers for Disease Control and Prevention—South Africa, Pretoria, South Africa; University of Hong Kong, HONG KONG

## Abstract

**Introduction:**

Data on the burden and risk groups for influenza-associated mortality from Africa are limited. We aimed to estimate the incidence and risk-factors for in-hospital influenza-associated severe acute respiratory illness (SARI) deaths.

**Methods:**

Hospitalised patients with SARI were enrolled prospectively in four provinces of South Africa from 2009–2013. Using polymerase chain reaction, respiratory samples were tested for ten respiratory viruses and blood for pneumococcal DNA. The incidence of influenza-associated SARI deaths was estimated at one urban hospital with a defined catchment population.

**Results:**

We enrolled 1376 patients with influenza-associated SARI and 3% (41 of 1358 with available outcome data) died. In patients with available HIV-status, the case-fatality proportion (CFP) was higher in HIV-infected (5%, 22/419) than HIV-uninfected individuals (2%, 13/620; p = 0.006). CFPs varied by age group, and generally increased with increasing age amongst individuals >5 years (p<0.001). On multivariable analysis, factors associated with death were age-group 45–64 years (odds ratio (OR) 4.0, 95% confidence interval (CI) 1.01–16.3) and ≥65 years (OR 6.5, 95%CI 1.2–34.3) compared to 1–4 year age-group who had the lowest CFP, HIV-infection (OR 2.9, 95%CI 1.1–7.8), underlying medical conditions other than HIV (OR 2.9, 95%CI 1.2–7.3) and pneumococcal co-infection (OR 4.1, 95%CI 1.5–11.2). The estimated incidence of influenza-associated SARI deaths per 100,000 population was highest in children <1 year (20.1, 95%CI 12.1–31.3) and adults aged 45–64 years (10.4, 95%CI 8.4–12.9). Adjusting for age, the rate of death was 20-fold (95%CI 15.0–27.8) higher in HIV-infected individuals than HIV-uninfected individuals.

**Conclusion:**

Influenza causes substantial mortality in urban South Africa, particularly in infants aged <1 year and HIV-infected individuals. More widespread access to antiretroviral treatment and influenza vaccination may reduce this burden.

## Introduction

Data on the burden and risk factors for influenza-associated mortality are key to guide targeted influenza vaccination programmes. This is particularly important in resource-limited settings where influenza vaccine availability is limited. However, data on influenza-associated mortality from African countries are scanty[1–4].

In South Africa, it is estimated from ecological modelling studies that influenza is responsible for approximately 2500 pneumonia and influenza-associated deaths each year in individuals of all ages[2, 5]. These studies indicate that the highest mortality associated with influenza is amongst children aged <5 years, HIV-infected adults aged 20–44 years and individuals aged ≥65 years. Validation of estimates from ecologic models is vital if such estimates are to be accepted and applied by policy makers. However, validation represents a challenge, as there are few gold-standard data that can be used for comparison. It has been suggested that, ideally, reference-standard results would be derived from prospective studies enrolling and testing hospitalized patients with a sensitive and specific laboratory test, such as polymerase chain reaction[6].

We aimed to estimate the incidence of influenza-associated severe acute respiratory illness (SARI) deaths and describe the risk-factors associated with death using data from prospective, hospital-based sentinel surveillance in South Africa.

## Methods

### Ethical considerations

The protocol was approved by the Research Ethics Committees of the Universities of the Witwatersrand (reference number M081042) and KwaZulu-Natal (reference number BF157/08). This surveillance was deemed non-research by the U.S. CDC and did not need human subjects review by that institution. All participants provided written informed consent to participate in the study.

### Surveillance programme

From February 2009 through December 2013, active, prospective, hospital-based surveillance for SARI was implemented in three of the nine provinces of South Africa (Chris Hani-Baragwanath Academic Hospital (CHBAH) in an urban area of Gauteng Province, Edendale Hospital in a peri-urban area of KwaZulu-Natal Province and Matikwana and Mapulaneng Hospitals in a rural area of Mpumalanga Province). In June 2010, an additional surveillance site was introduced at Klerksdorp and Tshepong Hospitals in a peri-urban area of the Northwest Province[7].

### Case definition

A case of SARI was defined as a hospitalised individual with illness onset within seven days of admission meeting age-specific inclusion criteria. We included children aged two days through <3 months with physician-diagnosed sepsis or acute lower respiratory tract infection (ALRI), children aged three months through <5 years with physician-diagnosed ALRI (including, for example bronchitis, bronchiolitis, pneumonia and pleural effusion) and patients aged ≥5 years meeting a modified World Health Organization (WHO) case definition for severe acute respiratory illness: (1) sudden onset of fever (>38°C) or reported fever, (2) cough or sore throat, and (3) shortness of breath, or difficulty breathing[8].

### Study procedures

All patients admitted during Monday through Friday were eligible, except for adult patients at CHBAH where enrolment occurred for two of every five working days (selected days varied systematically) per week due to large patient numbers and limited resources. In 2013, enrolment at CHBAH was down-scaled: paediatric patients were then enrolled on 2 of the 5 working days and adult patients on 1 of the 5 working days. Numbers of patients admitted, numbers meeting study case definitions and numbers enrolled were collected. Demographics, socio-economic factors, medical history, clinical presentation and outcome were recorded by means of interview and hospital record review. Study staff completed case report forms until discharge and collected respiratory (nasopharyngeal [NP] and throat swabs from patients aged ≥5 years or NP aspirates from patients aged <5 years) and blood specimens from consenting patients. Hospital and intensive care unit (ICU) admission and collection of specimens for bacterial culture, tuberculosis testing and CD4+ T-cell counts were performed according to attending-physician discretion. All patients enrolled into SARI surveillance were monitored until discharge or death to determine in-hospital outcome. Patients were not followed for outcome following discharge from hospital.

HIV-infection status was obtained based on testing undertaken as part of standard-of-care,[9] or through anonymised linked dried blood spot specimen testing by HIV polymerase chain reaction (PCR) assay for children aged <18 months and by ELISA for individuals aged ≥18 months. CD4+ T-cell counts were determined by flow cytometry[10]. Patients were categorised into two immunosuppression categories: (1) no or mild immunosupression (CD4+ T-lymphocytes ≥200/mm^3^or equivalent age-appropriate CD4+ percentage for children aged <5 years), or (2) severe immunosuppression (CD4+ T-lymphocytes <200/mm^3^ or equivalent age-appropriate CD4+ percentage for children aged <5 years)[11].

Underlying medical conditions were defined as asthma, other chronic lung disease, chronic heart disease, liver disease, renal disease, diabetes mellitus, immunocompromising conditions excluding HIV infection, neurological disease or pregnancy and were considered absent if indicated in medical records or when there was no direct reference to that condition.

### Laboratory methods

Respiratory specimens were transported in viral transport medium at 4–8°C to the National Institute for Communicable Diseases (NICD) of the National Health Laboratory Services (NHLS) within 72 hours of collection. Respiratory specimens were tested by a multiplex real-time reverse-transcription PCR assay for 10 respiratory viruses (influenza A and B viruses, parainfluenza virus 1, 2 and 3; respiratory syncytial virus; enterovirus; human metapneumovirus; adenovirus and rhinovirus)[12]. Influenza positive specimens were subtyped using the U.S. Centers for Disease Control and Prevention (CDC) real-time reverse-transcription PCR protocol for characterisation of influenza virus[13]. *Streptococcus pneumoniae* was identified by quantitative real-time PCR detecting the *lytA* gene from whole blood specimens[14].

### Risk factors for death

We assessed risk factors for death among influenza-positive SARI patients from 2009 through 2013. Missing data among influenza-positive SARI patients were imputed using chained equations over 10 imputation runs. Variables included in the multiple imputation model were HIV-status, sex, in-hospital outcome, presence of underlying illness, ventilation, use of oxygen, duration of hospitalisation, duration of symptoms, receipt of antibiotics on admission and pneumococcal *lytA* PCR positivity. Data were missing for 24% (329/1376) of individuals on HIV status, 24% (331/1376) for pneumococcal PCR and 5% (72/1376) for antibiotics given on admission. For all other variables missing data were ≤2%. Variables potentially on the causal path to death such as intensive care unit (ICU) admission and mechanical ventilation were not evaluated in the model of risk factors for death, but used as predictors during multiple imputation. Because initiation of tuberculosis treatment (in the absence of laboratory confirmation) may be more likely in patients who appear sicker and only a small percentage of patients (<30%) were tested for tuberculosis, receipt of tuberculosis treatment was also not evaluated in the model of risk factors for death. Univariate and multivariable logistic regression analyses were performed after multiple imputation. Multivariable logistic regression models were evaluated, starting with all variables that were significant at p<0.1 on univariate analysis, and dropping non-significant factors with stepwise backward selection. All two-way interactions of the variable significant at the final additive model were evaluated. Two-sided p values <0.05 were considered significant throughout. Age group, hospital, duration of hospitalisation and year were defined as categorical variables in multiple levels. All other variables were defined as the presence or absence of the attribute. The statistical analysis was implemented using Stata version 12 (StataCorp Limited, Texas, United States of America).

### Calculation of mortality rate

Calculation of mortality rate was conducted at one surveillance site (CHBAH) from 2009–2012 where population denominator data were available. This hospital is the only public hospital serving a community of about 1.3 million persons in 2011of whom an estimated 10% have private medical insurance[15]. Most (>80%) uninsured persons and approximately10% of insured persons seek care at public hospitals; consequently, we assumed that most persons requiring hospitalisation from this community are admitted to CHBAH.

To estimate the number and rate of in-hospital deaths associated with influenza for the period 2009–2012 (2013 was not included as enrolment was down-scaled in this year), we first estimated the age-specific (<1 year, 1–4 years, 5–24 years, 25–44 years, 45–64 years and > = 65 years) number of hospitalisations for SARI. We also separately estimated the rate of death for children aged <6 months as this age group could be potentially targeted through maternal influenza immunization. We used numbers of enrolled SARI patients and adjusted for non-enrollment in three of five adult wards and during weekends as well as refusal to participate using information from study logs. We then multiplied the SARI hospitalisations by the age-specific influenza detection ratio and the case-fatality proportion (CFP) amongst patients with influenza to obtain the estimated number of deaths in patients hospitalized with influenza. To obtain the number of HIV-specific influenza—associated deaths we assumed that the HIV prevalence was similar amongst influenza-positive patients who died and were tested for HIV and those not tested. Because in South Africa a large proportion of deaths occurs outside of hospital, we used the age-specific proportion of in-hospital deaths among individuals that died of pneumonia and influenza (International Classification of Diseases, 10^th^ revision [ICD-10] code: J10-J18) from vital statistics data to estimate the age-specific number of influenza-associated deaths occurring out-of hospital[16]. We chose to use pneumonia and influenza deaths because these are the ICD-10 codes most comparable to individuals with SARI, and we assumed that the proportion of in-hospital (vs out-of hospital) influenza-associated SARI deaths was similar to those of pneumonia and influenza. In 2009 (the most recent year for which vital statistics data were available), 29% of pneumonia and influenza deaths in Gauteng province (where CHBAH is located) occurred outside of the hospital (ranging from 24%-49% depending on the age group)[16]. We obtained the rate of influenza-associated SARI deaths per 100,000 person-years by age groups and HIV status using the estimated number of influenza-associated deaths (in and out of hospital) by HIV status divided by the mid-year population estimates for region D of Soweto, multiplied by 100,000[17]. The age- and year—specific HIV prevalence in the study population was obtained from the projections of the Actuarial Society of South Africa AIDS and Demographic model[18]. Confidence intervals for incidence estimates were calculated using the Poisson distribution. Age-specific and overall age-adjusted risk of influenza associated deaths in HIV-infected and-uninfected persons was determined using log-binomial regression.

## Results

### Patients enrolled and influenza seasonality

**Fig 1:**
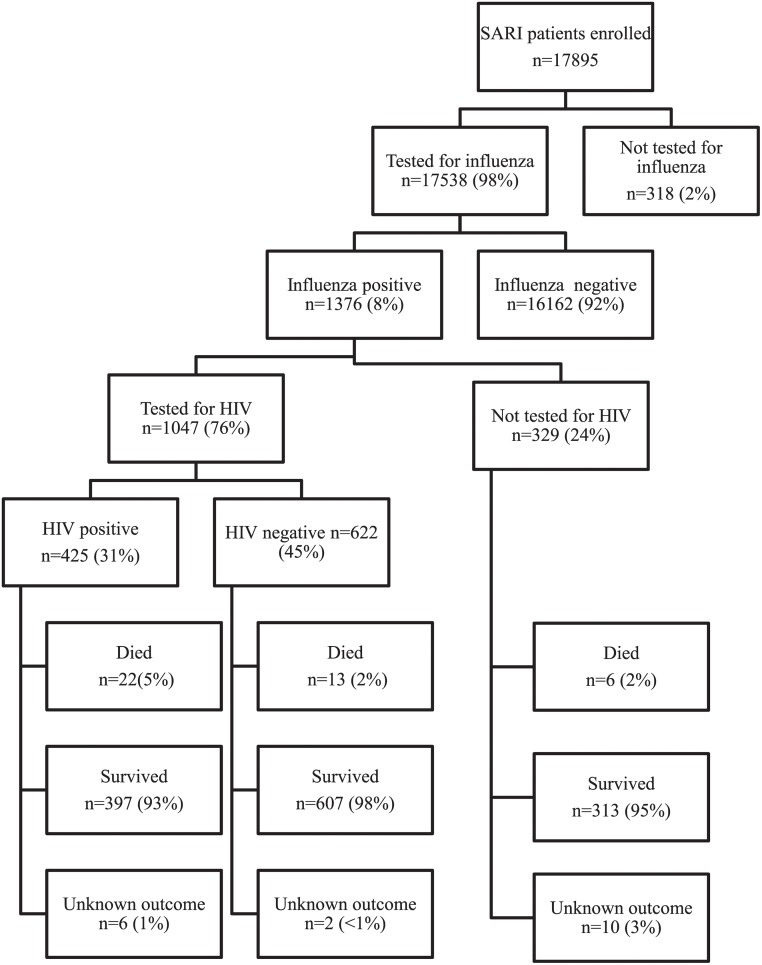
Flow chart of patients enrolled in the South African severe acute respiratory illness surveillance programme at four sites in South Africa, 2009–2013. HIV—human immunodeficiency virus.

From February 2009 through December 2013 we enrolled 17,895 individuals with SARI, of these 17,538 (98%) were tested for influenza and 1376 (8%) tested influenza positive (Fig. 1). The majority of patients (12,353/17,895, 69%) were enrolled at CHBAH. In-hospital outcome data was available for 99% (1358/1376) of influenza-positive individuals.

Amongst patients testing influenza positive, HIV test results were available for 68% (474/696) of children <5 years and 84% (573/680) of individuals ≥5 years and the HIV-prevalence was 11% (53/474) among children <5 years and 65% (372/573) in individuals ≥5 years. Underlying illnesses other than HIV were present in 3% (21/695) of children <5 years and 12% (83/675) of individuals ≥5 years. There were only 7 pregnant women with influenza-associated SARI enrolled, none of whom died.

Blood or pleural fluid specimens were submitted for culture from 286 individuals with SARI, 7 specimens were positive for bacterial growth (4 *Streptococcus pneumoniae*, and one each for *Haemophilus influenzae*, *Staphylococcus aureus* and *Neisseria meningitidis*). None of these 7 individuals died. Less than one third (30%, 412/1376) of individuals were tested for tuberculosis. Of these, 9% (35/412) tested tuberculosis positive.

**Fig 2:**
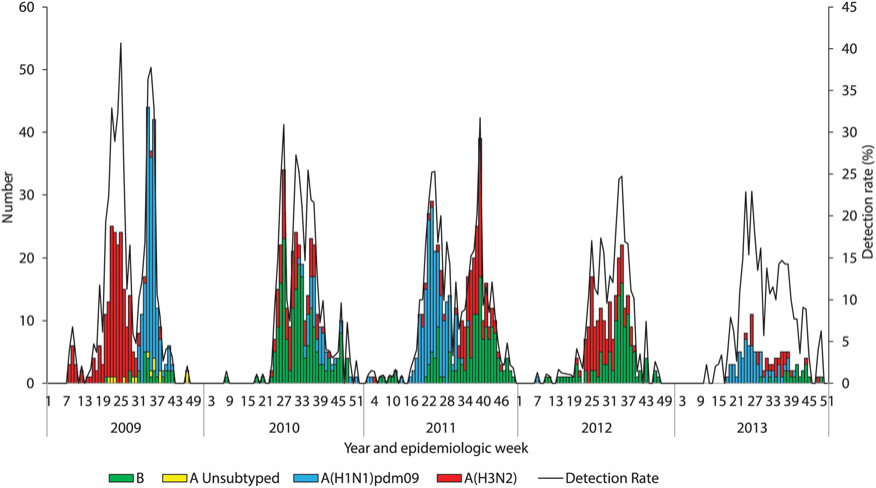
Number of patients testing influenza positive by subtype and influenza detection rate by epidemiologic week and year among patients hospitalized with severe acute respiratory illness at four sentinel surveillance sites, South Africa, 2009–2013.

In 2009, there was a peak in influenza A(H3N2), followed by a second peak of influenza A(H1N1)pdm2009. Influenza A(H1N1)pdm2009 was the most common subtype in 2011 (140/363, 39%) and 2013 (66/113, 58%), influenza B predominated in 2010 (165/274, 60%) and in 2012 influenza B (118/223, 53%) and influenza A(H3N2) co-circulated (Fig. 2).

### Characteristics of influenza-positive patients who died and factors associated with death

**Fig 3:**
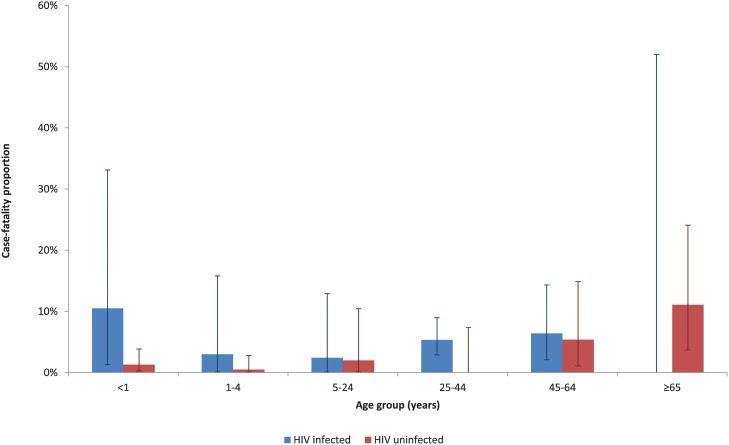
Case-fatality proportions by age group and HIV status amongst patients hospitalized with influenza-associated SARI at four sentinel surveillance sites in South Africa, 2009–2013 (n = 1039).

Among influenza-positive SARI patients from 2009 through 2013 with available data on in-hospital outcome, the overall in-hospital CFP was 3% (41/1358). The median time from hospital admission to death was 4 days (interquartile range (IQR) 1–15) and was longer in HIV-infected individuals (6 days, IQR 1–18) as compared to HIV-uninfected individuals (3 days, IQR 1–13; p<0.001). CFPs varied by age group, and among children were highest in children <1 year; and increased with increasing age amongst individuals >5 years (Table 1). All the children who died in the age group <1 year, were aged <6 months (CFP 3%, 6/178). The CFP varied by HIV status and was 5% (22/419) in HIV-infected individuals, 2% (13/620) (p = 0.006) in HIV-uninfected individuals and 2% (6/319) in those with unknown HIV status (Table 1). Amongst HIV-uninfected individuals, CFP was highest in individuals aged ≥65 (11%, 5/45) years, while in HIV-infected individuals the point estimates for CFP were highest in children <1 year (11%, 2/19) and adults aged 45–64 years (6%, 5/78; Fig. 3) but there was no statistically significant difference in CFP between the age groups. On multivariable analysis, factors independently associated with death were age-group 45–64 years (odds ratio (OR) 4.0, 95% confidence interval (CI) 1.01–16.3) and ≥65 years (OR 6.5, 95% CI 1.2–34.4) compared to 1–4 years, HIV-infection (OR 2.9, 95% CI 1.1–7.8), presence of underlying medical conditions (OR 2.9, 95% CI 1.2–7.3) and pneumococcal co-infection identified by whole blood LytA PCR (OR 4.1, 95% CI 1.5–11.2).

**Table 1:** Factors associated with death amongst hospitalised patients with influenza-associated severe acute respiratory illness at four sentinel surveillance sites, South Africa, 2009–2013.

Characteristics		Hospitalised case-fatality proportion (%)	Univariate analysis		Multivariable analysis^a^	
			OR (95% CI)	p	OR (95% CI)	p
**Demographic characteristics**						
Age group (years)	<1	6/351 (2)	1.9 (0.5–7.8)	<0.001	1.5 (0.3–7.0)	<0.001
	1–4	3/339 (1)	Reference		Reference	
	5–24	2/130 (2)	1.7 (0.3–10.6)		1.2 (0.2–7.4)	
	25–44	14/332 (4)	5.0 (1.4–17.5)		2.2 (0.5–8.8)	
	45–64	11/154 (7)	8.6 (2.4–31.3)		4.0 (1.0–16.3)	
	≥65	5/52 (10)	12.4 (2.9–53.9)		6.5 (1.2–34.4)	
Sex	Female	19/762 (2)	Reference	0.200		
	Male	22/596 (4)	1.5 (0.8–2.9)			
Race	Black African	40/1327 (3)	Reference	0.343		<0.001
	Other race	0/29 (0)	Undefined			
Site	CHBAH	28/940 (3)	Reference	0.796		
	Matikwana/ Mapulaneng	6/231 (3)	0.9 (0.4–2.2)			
	Edendale	5/111 (5)	1.6 (0.6–4.2)			
	Klerksdorp/Tshepong	2/76 (3)	0.9 (0.2–3.8)			
Year	2009	4/387 (1)	Reference	<0.001		
	2010	20/274 (7)	6.7 (2.3–19.8)			
	2011	5/361 (1)	1.2 (0.3–4.5)			
	2012	8/225 (4)	3.1 (0.9–10.4)			
	2013	4/111 (4)	3.3 (0.8–13.7)			
**Co-infections and underlying medical conditions**						
HIV status	Negative	13/620 (2)	Reference	0.006	Reference	0.034
	Positive	22/419 (5)	3.0 (1.5–6.0)		2.9 (1.1–7.8)	
Underlying medical condition^b^	No	31/1247 (2)	Reference	0.001	Reference	0.021
	Yes	9/110 (8)	3.3 (1.5–7.1)		2.9 (1.2–7.3)	
Smoking (≥12 years)	No	28/535 (5)	Reference	0.930		
	Yes	4/73 (5)	1.1 (0.4–3.1)			
Alcohol (≥12 years)	No	27/524 (5)	Reference	0.761		
	Yes	5/84 (6)	1.2 (0.5–3.3)			
Receiving tuberculosis treatment^c^ ^d^	No	30/1233 (2)	Reference	<0.001		
	Yes	11/125 (9)	3.9 (1.9–7.9)			
Laboratory-confirmed tuberculosis	No	8/369 (2)	Reference	0.197		
	Yes	2/35 (6)	2.7 (0.6–13.4)			
Pneumococcal coinfection^e^	No	23/946 (2)	Reference	0.003	Reference	0.006
	Yes	7/89 (8)	3.5 (1.4–8.4)		4.1 (1.5–11.2)	
Viral co-infection^f^	No	24/773 (3)	Reference	0.832		
	Yes	17/585 (3)	1.0 (0.5–1.8)			
Influenza type and subtype	H1N1pdm	7/398 (2)	Reference	0.103		
	H3N2	14/490 (3)	1.5 (0.6–3.8)			
	B	19/447 (4)	2.3 (0.9–5.6)			
Receipt of pneumococcal conjugate vaccine^g^	No	2/17 (1)	Reference	0.594		
	Yes	1/161 (1)	0.4 (0.1–4.6)			
**Clinical presentation and course**						
Duration of symptoms prior to admission	< 2 days	7/381 (2)	Reference	0.171		
	≥ 2 days	31/966 (3)	1.8 (0.8–4.1)			
Antibiotics prescribed on admission	No	2/49 (4)	Reference	0.712		
	Yes	37/1247 (3)	0.8 (0.2–3.3)			
Oxygen administered ^d^	No	15/898 (2)	Reference	<0.001		
	Yes	25/454 (6)	3.4 (1.7–6.5)			
Mechanical ventilation^d^	No	38/1343 (3)	Reference	0.001		
	Yes	2/9 (22)	10.5 (2.1–51.7)			
Admitted to intensive care unit^d^	No	39/1341 (3)	Reference	0.228		
	Yes	1/11 (9)	3.3 (0.4–26.7)			
Duration of hospitalisation (days)	<2	4/323 (1)	Reference	0.001		
	2–7	18/726 (2)	2.0 (0.7–6.0)			
	>7	18/290 (6)	5.3 (1.8–15.8)			

OR—Odds ratio, CI—confidence interval, HIV—human immunodeficiency virus.

^a^ Odds ratios and p values shown for all variables included in the multivariable model. For the multivariable analysis of factors associated with death we did not evaluate or include variables which may be on the continuum to the outcome (ICU admission or receipt of mechanical ventilation or oxygen therapy) in the multivariable model.

^b^ Asthma, other chronic lung disease, chronic heart disease (valvular heart disease, coronary artery disease, or heart failure excluding hypertension), liver disease (cirrhosis or liver failure), renal disease (nephrotic syndrome, chronic renal failure), diabetes mellitis, immunocompromising conditions excluding HIV infection (organ transplant, immunosuppressive therapy, immunoglobulin deficiency, malignancy), neurological disease (cerebrovascular accident, spinal cord injury, seizures, neuromuscular conditions) or pregnancy. Comorbidities were considered absent in cases for which the medical records stated that the patient had no underlying medical condition or when there was no direct reference to that condition.

^c^ Receiving tuberculosis treatment or started on tuberculosis treatment during the course of the admission.

^d^ Not evaluated in the multivariable model.

^e^ Four additional cases of *S*. *pneumoniae* on blood culture not included.

^f^ Co-infection with at least one of parainfluenza virus 1, 2 and 3; respiratory syncytial virus; enterovirus; human metapneumovirus; adenovirus; rhinovirus in addition to influenza.

^g^ Children <5 years only.

CD4+ T cell count data was only available for 29% (122/425) of HIV-infected individuals. CFP was higher (16%, 12/72) in individuals with severe immunosuppresion (CD4+ T cell count <200/μl or age-specific equivalent) as compared to those without severe immunosuppresion (4%, 2/50; p = 0.012). Data on antiretroviral treatment (ART) was available for 59% (251/425) of HIV-infected individuals and 126 (50%) reported receiving ART. The CFP observed among those receiving ART (5%, 6/133) was not statistically different than those not receiving ART (7%, 8/113; p = 0.386).

### Rate of influenza-associated SARI deaths

The estimated rate of influenza-associated SARI deaths per 100,000 person-years at one site (CHBAH) was 4.7 (95% CI: 4.1–5.3) and was highest in children <1 year (20.1, 95% CI 12.1–31.3) and adults aged 45–64 years (10.4, 95% CI 8.4–12.9; Table 2). The rate of influenza-associated SARI deaths was higher in HIV-infected than-uninfected individuals in all age groups except for ≥65 years where no HIV-infected individuals who died were identified. Adjusting for age, the rate of SARI death was 20.4 (95% CI 15.0–27.8) times higher in HIV-infected than HIV-uninfected individuals.

**Table 2:** Incidence of mortality for influenza-associated severe acute respiratory illness per 100,000 person years by HIV status in Soweto, South Africa, 2009–2013.

Age group (years)	All in- and out-of-hospital N	HIV infected in- and out-of-hospita N	HIV uninfected in- and out-of-hospital N	All in-hospital Rate/100,00 person years (95% CI)	All out-of-hospital Rate/100,00 person years (95% CI)	All in- and out-of-hospital Rate/100,00 person years (95% CI)	HIV infected in- and out-of-hospital Rate/100,00 person years (95% CI)	HIV uninfected in- and out-of-hospital Rate/100,00 person years (95% CI)	HIV infected vs HIV uninfected in- and out-of-hospital Relative risk (95% CI)
<1	18	13	6	10.6 (5.1–19.4)	8.5 (3.7–16.7)	20.1 (12.1–31.3)	368.3 (190.3–643.4)	6.6 (2.4–14.3)	56.1 (19.5–182.1)
1–4	14	8	8	1.8 (0.7–3.7)	1.8 (0.7–3.7)	3.9 (2.2–6.4)	39.5 (15.9–81.4)	1.9 (0.8–3.9)	20.9 (6.3–69.9)
5–24	9	4	4	0.3 (0.1–0.7)	0.2 (0.03–0.5)	0.5 (0.2–0.9)	4.9 (1.6–11.4)	0.3 (0.1–0.6)	17.3 (4.0–75.1)
25–44	92	92	0	3.9 (3.1–5.0)	1.4 (0.9–2.0)	5.3 (4.3–6.0)	20.3 (16.3–24.9)	0.0 (0.0–0.3)	Not estimated
45–64	86	54	33	8.0 (6.2–10.2)	2.4 (1.5–3.8)	10.4 (8.4–12.9)	51.2 (38.5–66.8)	4.5 (3.1–6.3)	11.5 (7.3–18.4)
≥65	23	0	23	6.0 (3.4–9.7)	2.6 (1.1–5.4)	8.2 (5.2–12.5)	0 (0–103.6)	8.4 (5.2–12.7)	Not estimated
<5	34	19	13	3.7 (2.2–5.9)	3.3 (1.9–5.4)	6.8 (4.7–9.6)	95.3 (58.2–147.2)	2.8 (1.5–4.8)	33.9 (16.1–74.2)
≥5	209	150	59	3.2 (2.7–3.7)	1.2 (0.9–1.6)	4.5 (3.9–5.1)	22.5 (19.1–26.5)	1.5 (1.1–1.9)	15.4 (11.3–21.2)
All ages	243	170	73	3.3 (2.8–3.8)	1.4 (1.1–1.8)	4.7 (4.1–5.3)	24.8 (21.2–28.8)	1.7 (1.3–2.0)	20.4 (15.0–27.8)*

N—estimated number of deaths CI—confidence interval, HIV—human immunodeficiency virus

*Age-adjusted.

The estimated rate of in- and out- of-hospital SARI deaths per 100,000 person years in children aged <6 months was 39.4 (95% CI 24.2–62.7) overall and 763.5 (95% CI 381.2–1283.4) in HIV-infected children and 13.6 (95% CI 4.8–28.6) in HIV-uninfected children. The rate of death per 100,000 person years in children <6 months was 21.8 (95% CI 10.1–38.9) for in-hospital deaths and 17.6 (7.3–33.3) for out-of-hospital deaths.

## Discussion

We have documented that influenza causes substantial mortality in Soweto, South Africa. The peak burden of mortality is experienced in children aged <1year of age (particularly those aged <6 months) and HIV-infected adults aged 25–64 years. HIV-infected individuals experienced a higher estimated rate of death in all age groups. Other risk factors for death were the presence of non-HIV underlying illness and co-infection with *S*. *pneumoniae*.

Based on our data, age is an important risk factor for influenza-associated death, with the highest estimated rates of death were highest in the <1 year age group and then generally increased with increasing age amongst older individuals. This is similar to finding from the USA where mortality increases with increasing age and is higher in <1 year compared to 1–4 year olds[19].

Amongst children aged <5 years we estimated 6.8 influenza-associated SARI deaths per 100,000 person-years (95% CI 4.7–9.6) in Soweto. This is similar to estimates of influenza-associated mortality among pneumonia and influenza deaths from South African ecological modelling studies (7 per 100,000 person-years, 95% CI 4–11)[2] but higher than estimates from ecological studies in the United States of America (0.2–0.3 per 100,000 person-years)[19]. A community-based study from Bangladesh found substantially lower rates of influenza-associated respiratory deaths (1.5 per 100,000 person-years 95% CI 0.9–2.0) in children <5 years of age[20]. Among children <5 years of age, rates of in-hospital influenza-associated deaths per 100,000 person-years from hospital-based SARI surveillance were 7.6 (95% CI 2.1–13.2) in Kenya compared to 3.7 (95% CI: 2.2–5.9) in South Africa but the proportion of out-of hospital deaths was not reported, limiting comparability with our study[21].

Amongst individuals ≥5 years we estimated 4.5 respiratory deaths per 100,000 person-years (95% CI 3.9–5.1) in the Soweto area. This is similar to estimates of influenza-associated mortality among pneumonia and influenza deaths (5.2 per 100,000 person-years; 95% CI 2.4–6.1) from ecological modelling studies from South Africa[5]. Estimates of influenza-associated mortality from our study were similar to estimates from modelled ecologic data in South Africa in all age groups (confidence intervals overlapped), except in elderly individuals where estimates in our study were substantially lower (8.2 deaths per 100,000 person-years, 95% CI 5.2–12.5 in persons aged ≥65 years) compared to ecological data (20.8 deaths per 100,000 person-years, 95% CI 12.5–29.8 in persons aged 65–74 years and 83.0 deaths per 100,000 person-years, 95% CI 57.8–1 in persons aged ≥75 years)[2, 5]. This may reflect the fact that elderly individuals are less likely to present to hospital and/or to be enrolled in surveillance, however, there are no published data evaluating health-seeking behaviour in the elderly in South Africa. A study from Kenya found similarly low mortality rates in the elderly based on hospitalisation data, likely as a result of low levels of hospital utilisation in this group[22].

Overall, in our study, HIV-infected individuals had ~20 times greater estimated rates of mortality than HIV-uninfected individuals. An analysis of data from the same surveillance programme found that the relative risk of influenza-associated hospitalisation was 4–8 times higher in HIV-infected compared to HIV-uninfected individuals, somewhat lower than the relative risk for influenza-associated mortality described in this study. This is likely because the elevated mortality rates in our study reflect both the elevated risk of hospitalisation in HIV-infected individuals, as well as the elevated CFP once hospitalised[7]. The increased CFP could reflect the presence of co-infections such as tuberculosis and pneumococcus in HIV-infected individuals, or might be as a direct result of influenza infection. Ecological studies from South Africa estimate ~10 times elevated mortality risk in HIV-infected individuals[2, 5]. These slightly lower relative risks than those found in our study are likely driven by the higher rates of estimated influenza-associated deaths in the elderly (who are predominantly HIV-uninfected) from ecological studies. Rates of deaths were elevated in HIV-infected individuals for all age groups except for elderly individuals aged ≥65 years where numbers of cases were very small and we did not identify any HIV-infected influenza-positive individuals who died. This finding is similar to estimates from ecological studies of influenza-associated mortality from South Africa, where rates of death were higher in HIV-infected individuals of all age groups except in the elderly where no influenza-associated deaths were estimated in HIV-infected individuals[2, 5]. A case-series of deaths due to influenza A(H1N1)pdm09 in South Africa found that 53% were HIV-infected[23]. Studies amongst children at a single hospital in South Africa found an elevated incidence of influenza-associated hospitalisation in HIV-infected children but CFPs were similar in HIV-infected and—uninfected groups[24]. Two reviews of the published data on influenza epidemiology in HIV-infected individuals found that HIV-infected individuals experience higher rates of influenza-associated mortality as compared to HIV-uninfected individuals especially at low CD4+ T cell counts[25, 26]. Similarly, we found elevated CFP in individuals with low CD4+ T cell counts. Some published studies have found similar mortality in HIV-infected to HIV-uninfected individuals, but in most of these studies the vast majority of individuals were receiving ART[25, 27]. Studies using both individual-level as well as ecological data suggest that more widespread access to ART will likely reduce the mortality burden associated with HIV in South Africa[3, 25–27].

We identified underlying illness (other than HIV) as an important risk factor for death, similar to findings from other studies^28–30^. The prevalence of underlying illness (other than HIV) in our study was, however, lower than has been described in other settings; this may be partly as a result of under-ascertainment of underlying conditions and partly related to the high relative contribution of HIV[7, 27–29].

Pneumococcal co-infection was independently associated with increased risk of death in this group of patients hospitalised with influenza. Bacterial co-infection is a well described risk factor for severe outcome amongst patients with influenza both for seasonal and pandemic influenza[30, 31]. We have previously shown that the prevalence of pneumococcal infection is elevated in HIV-infected individuals hospitalised with influenza-associated SARI in South Africa [7]. In addition, we found that both underlying HIV and influenza infection are independent risk factors for increased pneumococcal load in the blood[32]. Increased pneumococcal load is, in turn, associated with increased mortality. Nonetheless, real time PCR is a sensitive method for detecting the pneumococcus in the blood[32], We cannot exclude that the detection of *lytA* may reflect transient bacteraemia in some individuals[33].

This study included estimates of mortality from seasonal influenza virus as well as influenza A(H1N1)pdm09 in 2009 in evaluation of mortality rates and factors associated with death. In a previously published analysis from South Africa from the same surveillance programme, CFPs were similar for influenza A(H1N1)pdm09 and influenza A(H3N2) and influenza B and the only difference in epidemiology between the different types and subtypes was the age-distribution with a younger age of hospitalisation associated with influenza A(H1N1)pdm09[34]. Studies have shown that, similar to seasonal influenza, CFPs for influenza A(H1N1)pdm09 increased with increasing age even though rates of hospitalisation were elevated in younger age groups[29, 35]. We did not have sufficient numbers of deaths to separately evaluate rates of mortality or risk factors for death for influenza A(H1N1)pdm09.

This study has several potential limitations. The number of deaths identified through surveillance was relatively low (n = 41). This reduced our power to identify additional potential factors associated with increased risk of mortality and affected the precision of our rate estimates, especially in some age subgroups. Additional uncertainty may have been introduced in the process of extrapolation from in-hospital deaths to mortality burden. This was not included in the estimation of confidence intervals. Individuals who died may have been less likely to be enrolled and have available HIV status data as they were unable to provide consent. This could have potentially biased our findings. Our estimates of mortality rates assumed that all patients in Soweto access care at CHBAH hospital, while some may access private care. In addition, we only evaluated respiratory deaths, however influenza-associated deaths due to non-respiratory causes may be substantial. Therefore, our estimates likely represent a minimum estimate. Nevertheless, the estimates of relative risk by HIV status should be robust, unless patients had differential access to care by HIV-infection status[27]. We extrapolated the numbers of deaths occurring outside of the hospital using vital registration data from Statistics South Africa for Gauteng Province from 2009 under the assumption that these data were representative of Soweto[16]. Vital registration data in this year was >90% complete and location of death is not subjective and therefore was likely correctly coded.

Pregnant woman are a well-described risk group for mortality for both seasonal and pandemic influenza, and pregnant women are a priority group for influenza vaccination according to World Health Organisation recommendations[29, 36–38]. Numbers of pregnant women were low in our study, likely because review of maternity ward admissions was not consistently performed and numbers of pregnant women hospitalised with influenza are low[7]. Data on influenza-associated mortality amongst pregnant women from Africa are scanty and are urgently needed to guide policy related to vaccination of this group[1, 39].

In conclusion, we have demonstrated a substantial burden of influenza-associated mortality in South Africa, particularly in infants <1 year and HIV-infected individuals. More widespread access to ART may reduce the mortality burden in HIV-infected individuals[3]. Influenza vaccination programmes targeting HIV-infected individuals as well as pregnant women (particularly aiming to reduce influenza mortality burden in their infants) may also reduce influenza-associated mortality.
